# Omission of Sentinel Lymph Node Biopsy in Breast Cancer: A Real‐World Validation of the Patient Populations of the SOUND and INSEMA Trials

**DOI:** 10.1002/jso.70256

**Published:** 2026-05-06

**Authors:** Tia Puiras, Eeva Juhanoja, Anselm Tamminen

**Affiliations:** ^1^ Faculty of Medicine University of Turku Turku Finland; ^2^ Department of Oncology Turku University Hospital Turku Finland; ^3^ Department of Plastic and General Surgery Turku University Hospital Turku Finland

**Keywords:** axillary lymph node dissection, breast cancer, de‐escalation, sentinel lymph node biopsy, surgery

## Abstract

**Background:**

Treatment guidelines recommending omission of axillary surgery in breast cancer are largely based on the SOUND and INSEMA trials. However, the extent to which their study populations represent real‐world patients remains unclear. We aimed to evaluate the real‐world applicability and external validity of these trial populations.

**Materials and Methods:**

All consecutive patients treated for early breast cancer at a single university hospital between 2010 and 2018 were included. Patients with clinically node‐negative disease were identified, and eligibility according to the SOUND and INSEMA inclusion criteria was determined. Clinicopathologic characteristics were compared between trial‐eligible real‐world patients and published trial populations.

**Results:**

A total of 2787 consecutive patients with clinically negative axilla were included; 71% (1982/2787) fulfilled the INSEMA and 52% (1461/2787) the SOUND trial eligibility criteria. Patients eligible in the SOUND trial were largely representative of real‐world patients in terms of clinicopathologic characteristics. In contrast, the INSEMA trial appeared more selected, with a higher proportion of biologically favorable tumors. Both trials predominantly included patients with small (< 2 cm) luminal breast cancers. Patients with larger tumors and more aggressive subtypes were underrepresented.

**Conclusion:**

The SOUND and INSEMA eligibility criteria are broadly applicable to real‐world patients with small luminal breast cancers. However, differences between trial populations and real‐world patients highlight the need for careful consideration when applying SLNB omission beyond these lower‐risk subgroups.

## Summary

The SOUND and INSEMA trials support the omission of sentinel lymph node biopsy in small luminal breast cancers, but their study populations do not fully reflect real‐world patients. Comparison with a large university hospital cohort showed that early‐stage cases are particularly overrepresented in INSEMA, limiting generalizability, whereas the SOUND trial resembles real‐world data in most aspects.

## 1 Introduction

Breast cancer is the most commonly diagnosed cancer among women with 2.3 million new cases in 2022, accounting for 23.8% of all cancer cases in females worldwide. It is the leading cause of cancer mortality in women with an estimated 666 000 deaths in 2022, accounting for 15.4% of all female cancer‐related deaths [1].

The nodal status of the axilla is an important prognostic factor for breast cancer. The pathological status of a sentinel lymph node (SLN) is known to accurately represent the pathological nodal status in the axilla, and implementation of sentinel lymph node biopsy (SLNB) has revolutionized axillary staging [2]. SLNB has largely replaced ALND as the standard method for axillary staging in clinically node‐negative (cN0) breast cancer [3]. Studies have shown that in patients with a SLN negative breast cancer, ALND can be safely omitted without compromising overall survival or recurrence outcomes [4]. Furthermore, randomized clinical trials (RCTs) such as the ACOSOG Z0011 trial, AMAROS, and SENOMAC have demonstrated that selected patients with limited axillary disease can reach sufficient regional control with adjuvant therapies, suggesting redundancy of ALND [5, 6, 7, 8, 9]. Recently, even the necessity of SLNB in selected patients has been questioned, particularly following the publication of the SOUND and INSEMA trials. These trials demonstrated the oncologic safety of omitting SLNB in carefully selected patients with cN0 early breast cancer and represent a major step toward de‐escalation of axillary surgery. The results have already influenced international treatment guidelines [10, 11, 12, 13].

Although RCTs are the cornerstone of evidence‐based medicine, it is critically important to assess how well their findings apply to unselected real‐world patients and clinical settings. Clinical trials are conducted under highly controlled conditions and often include carefully selected patient populations, which may result in selection toward lower‐risk patients. Therefore, their external validity—or generalizability—must be evaluated in relation to actual clinical practice. If the patients included in a clinical trial do not adequately represent those to whom treatment guidelines are applied, translating the results into practice may carry significant risks. Consequently, it is essential to validate the trial populations with real‐world data to identify potential selection biases and ensure that clinical guidelines are not applied either too broadly or too narrowly [14].

The aim of this study was to evaluate the real‐world applicability and external validity of the SOUND and INSEMA eligibility criteria by comparing the clinicopathologic characteristics of eligible patients in a large consecutive real‐world cohort treated at a single university hospital, thereby providing clinically relevant context for the implementation of these trial findings in routine practice.

## 2 Materials and Methods

Data from all consecutive patients who underwent surgery for invasive breast cancer at single tertiary referral hospital between 2010 and 2018 were analyzed. The data were retrieved from the Clinical Informatics Register of Auria Biobank and in case of discrepancies in the data and in unclear situations, the information received from the Auria Biobank was also verified from the electronic patient records at Turku University Hospital. The study period was selected to reflect a consistent clinical practice in which ALND was routinely performed in patients with positive SLNB, allowing accurate assessment of axillary nodal burden. All patients underwent preoperative ultrasound examination of axilla. The treatment guidelines followed the national guideline set by the Finnish Breast Cancer Group [15].

All female patients with primary invasive unilateral cN0 breast cancer, and upfront surgical treatment were included. The patient was considered cN0 if no suspicious axillary lymph nodes were detected in clinical examination nor in axillary ultrasound, or if the biopsy of a suspicious lymph node was negative in pathological examination.

The following data were collected: the year of the operation, age of the patient, performed surgery, laterality, weight, height and related body mass index (BMI), ASA classification, smoking status, diabetes and histopathological results: histological type of cancer, estrogen receptor (ER) and progesterone receptor (PgR), HER2 status, proliferation index (Ki‐67), grade and multifocality. Regarding axillary surgery, the number of removed SLNs and metastatic lymph nodes was recorded, and in the case of axillary clearance, the number of removed non‐metastatic and metastatic lymph nodes was recorded for SLNB and, when performed, subsequent ALND. These characteristics were compared with published baseline characteristics of the SOUND and INSEMA trial populations. The data from these trials were collected between 2012 and 2017 and between 2015 and 2019, respectively.

### 2.1 Definition of SOUND and INSEMA Eligibility

Eligibility according to the SOUND and INSEMA trials was determined by applying the original trial inclusion criteria to our cohort based on available clinicopathologic data. Patients were classified as SOUND‐eligible if they had cN0 breast cancer, tumor size ≤ 2 cm, and fulfilled other relevant trial inclusion criteria as defined in the SOUND protocol [12]. Similarly, patients were classified as INSEMA‐eligible based on the published inclusion criteria, including cN0 breast cancer and eligibility for breast‐conserving surgery, as defined in the original INSEMA trial protocol [13].

All eligibility assessments were performed retrospectively using prospectively recorded clinical and pathological data.

### 2.2 Statistical Analysis

The distribution of patients' characteristics was evaluated, and frequency tables were created. For patients' age and BMI, median and interquartile ranges were determined. The Pearson *χ* ^2^ test was performed at frequency tables and related *p* value was reported. For variables where only summary statistics were available from published trial reports, formal statistical comparisons were not performed and descriptive comparisons were used. All the analyses were performed using JMP Pro 18 (SAS Institute, Cary, North Carolina, USA) analysis software.

The European Society for Medical Oncology (ESMO) GROW checklist was used to guide the design of this study.

## 3 Results

**Figure 1:**
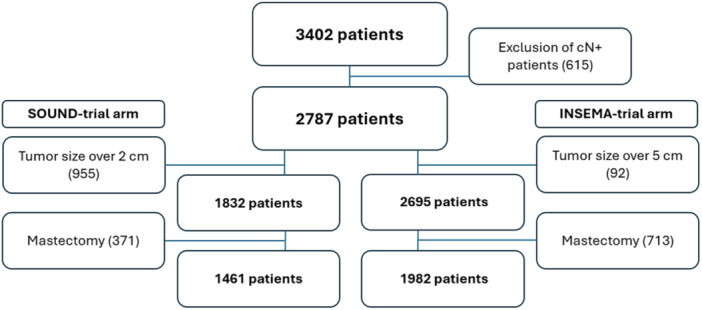
Flow chart presenting the exclusion of patients not fulfilling the criteria for sentinel lymph node biopsy arm of the SOUND and INSEMA trials.

The primary data study consisted of 3402 patients with invasive breast cancer. After excluding 615 patients with preoperatively detected axillary metastasis, 2787 patients remained eligible for analysis. After applying the inclusion criteria of the SOUND and INSEMA trials [12, 13], 1461 (52%) and 1982 (71%) patients, respectively, were eligible for further analyses (Figure 1).

The baseline clinicopathologic characteristics of the study cohort are presented in Table 1.

**Table 1:** Baseline patient characteristics of the real‐world data.

Number of patients	2787
Age—years, median (IQR)	64.2 (56.1–72.0)
BMI—kg/m^2^, median (IQR)	26.7 (23.3–30.8)
*ASA classification*	
ASA 1	491 (17.6)
ASA 2	1452 (52.1)
ASA 3	773 (27.7)
ASA 4	57 (2.0)
Unknown	15 (0.5)
*Histological type of breast cancer*	
Ductal	2225 (79.8)
Lobular	359 (12.9)
Other	203 (7.3)
*pT stage, TNM classification*	
pT1a	80 (2.9)
pT1b	531 (19.1)
pT1c	1221 (43.8)
pT2	863 (31.0)
pT3‐4	92 (3.3)
*pN stage, TNM classification*	
pN0	1979 (71.0)
pN1	679 (24.4)
pN2	95 (3.4)
pN3	34 (1.2)
*Multifocality*	
Yes	619 (22.2)
No	2168 (77.8)
*Estrogen receptor status (>* *0%)*	
Negative	221 (7.9)
Positive	2546 (91.4)
Unknown	20 (0.7)
*Progesterone receptor status (>* *0%)*	
Negative	304 (10.9)
Positive	2460 (88.3)
Unknown	23 (0.8)
Human epidermal growth factor 2 (HER2)	
Negative	2517 (90.3)
Positive	248 (8.9)
Unknown	22 (0.8)
Proliferation index (Ki‐67%)—median (IQR)	18 (10–30)
*Grade (histological* *+* *nuclear grade)*	
I	405 (14.5)
II	1586 (56.9)
III	775 (27.8)
Unknown	21 (0.7)
Invasion to blood vessels, lymphatic vessels, or nerves	176 (6.3)
*Laterality*	
Right	1388 (49.8)
Left	1399 (50.2)
*Surgery*	
Breast conserving surgery	1997 (71.7)
Mastectomy	790 (28.3)

*Note:* Values are *n* (%) unless otherwise specified.

Abbreviations: ASA = American Society of AnesthesiologistsI, BMI = body mass index, QR = interquartile range.

Overall, the real‐world cohort represented a typical early breast cancer population, with the majority of tumors being pT1 (65.8%) and hormone receptor–positive (91.4% ER‐positive). However, a substantial proportion of patients had higher‐risk features, including pT2–4 tumors (34.3%), Grade III tumors (27.8%), and nodal metastases (29.0%), reflecting the broader risk spectrum encountered in routine clinical practice.

The comparison of the SLNB‐arm of the SOUND‐trial and respective patients in the real‐world data is presented in Table 2 and the comparison of the SLNB arm of the INSEMA trial and respective patients in the real‐world data is presented in Table 3.

**Table 2:** Comparison between patient population of SOUND trial and real‐world data.

	SOUND eligible real‐world data	SLNB arm of the SOUND trial	*p*
Number of patients	1461	708	
*Age at surgery—years*			
< 40	14 (1.0)	10 (1.4)	< 0.001
40–49	115 (7.9)	114 (16.1)	
50–64	736 (50.4)	324 (45.8)	
≥ 65	596 (40.8)	260 (36.7)	
Median (IQR)	63 (57–69)	60 (52–68)	
*Histological type of breast cancer*			< 0.001
Ductal	1251 (85.6)	551 (77.8)	
Lobular	110 (7.5)	61 (8.6)	
Other (incl. tubular)	100 (6.8)	96 (13.5)	
*Pathological tumor size*			
pT1a	60 (4.1)	71 (10.0)	< 0.001
pT1b	460 (31.5)	251 (35.5)	
pT1c	941 (64.4)	355 (50.1)	
pT2	0 (0)	31 (4.4)	
Median (IQR), cm	1.2 (0.9–1.6)	1.1 (0.8–1.5)	
*Number of positive sentinel lymph nodes*			
0	1167 (79.9)	599 (84.6)	0.09
1	207 (14.2)	93 (13.1)	
≥ 2	50 (3.4)	14 (2.0)	
No sentinel lymph node biopsy	37 (2.5)	12 (1.7)	
*Number of positive lymph nodes*			
0	1196 (81.9)	599 (84.6)	0.033
1–3	242 (16.6)	93 (13.1)	
4–9	18 (1.2)	2 (0.3)	
≥ 10	5 (0.3)	2 (0.3)	
No information	0 (0)	12 (1.7)	
*Grade*			0.30
I	329 (22.5)	164 (27.7)	
II	853 (58)	377 (53.8)	
III	268 (18.3)	130 (18.5)	
No information	11 (0.8)	0 (0)	
*ER status*			0.052
0	83 (5.7)	56 (7.9)	
> 0	1361 (94.3)	652 (92.1)	
*PgR status*			< 0.001
0	117 (8.1)	108 (15.3)	
> 0	1333 (91.9)	600 (84.7)	
*HER2 status*			0.65
Not overexpressed	1345 (92.7)	660 (93.2)	
Overexpressed	105 (7.3)	48 (6.8)	
*Proliferation index (Ki‐67%)*			0.023
< 20	859 (59.3)	455 (64.4)	
≥ 20	590 (40.7)	252 (35.6)	
Median (IQR)	15 (9–25)	15 (10–23)	
*Surrogate subtype*			0.037
Luminal HER2‐negative	1291 (89.1)	617 (87.1)	
HER2‐enriched	105 (7.2)	48 (6.8)	
Triple‐negative	53 (3.7)	43 (6.1)	

Abbreviations: ER = estrogen receptor, HER2 = human epidermal growth factor receptor 2, IQR = interquartile range, PgR = progesterone receptor.

**Table 3:** Comparison of the INSEMA trial and the real‐world patient populations.

	INSEMA eligible real‐world data	SLNB arm of the INSEMA trial	*p*
Number of patients	1982	3896	
*Age at surgery (years)*			
< 35	1 (0.1)	6 (0.2)	< 0.001
35 to < 50	201 (10.1)	407 (10.4)	
50 to < 60	522 (26.3)	1278 (32.8)	
60 to < 70	807 (40.7)	1454 (37.3)	
≥ 70	451 (22.8)	751 (19.3)	
*BMI*			< 0.001
< 30 kg/m^2^	499 (67.4)	2913/3896 (74.8)	
≥ 30 kg/m^2^	241 (32.6)	983/3896 (25.2)	
*Pathological tumor size*			
pT0, pTis, or pTX	0 (0)	34 (0.9)	< 0.001
pT1	1461 (73.7)	3082 (79.1)	
pT2	521 (26.3)	756 (19.4)	
pT3–4	0 (0)	24 (0.6)	
*Nodal status—sentinel lymph nodes*			
pN0	1490 (75.2)	3275/3854 (85.0)	< 0.001
pN1mi	88 (4.4)	133 (3.5)	
pN1	336 (17.0)	438/3854 (11.4)	
pN2–3	68 (3.4)	8/3854 (0.2)	
Unknown	0 (0)	4	
*Nodal status—all removed lymph nodes*			
pN0	1490 (75.2)	50/253 (19.8)	< 0.001
pN1mi	88 (4.4)	1/253 (0.4)	
pN1	336 (17.0)	169/253 (66.8)	
pN2	50 (2.5)	33/253 (13.0)	
pN3	18 (0.9)		
*ER and PgR status*			< 0.001
Negative	118 (6.0)	58/3893 (1.5)	
Positive	1852 (94.0)	3835/3893 (98.5)	
Unknown	12	3	
*HER2 status*			< 0.001
Negative	1810 (91.3)	3755/3885 (96.7)	
Positive	161 (8.1)	130/3885 (3.3)	
Unknown	11	11	
*Intrinsic subtype*			< 0.001
HR positive, HER2 negative	1715 (86.5)	3705/3884 (95.4)	
HER2 positive	161 (8.1)	130/3884 (3.3)	
Triple‐negative breast cancer	94 (4.7)	49/3884 (1.3)	
Unknown	12		
*Tumor grade*			< 0.001
I	348 (17.6)	1463 (37.6)	
II	1156 (58.3)	2294 (58.9)	
III	465 (23.4)	139 (3.6)	
No information	13		
*Proliferation index (Ki‐67)*			
≤ 20%	1304 (65.8)	3220 (86.9)	< 0.001
> 20%	664 (33.5)	485/3705 (13.1)	
Unknown	14	191	
*Histological subtype*			< 0.001
Invasive carcinoma (ductal carcinoma, no special type)	1651 (83.3)	2828/3895 (72.6)	
Invasive or mixed lobular carcinoma	190 (9.6)	491/3895 (12.6)	
Other	141 (7.1)	576/3895 (14.8)	
Unknown	0	1	

Abbreviations: BMI = body mass index, ER = estrogen receptor, HER2 = human epidermal growth factor receptor 2 HR = hormone receptor, PgR = progesterone receptor.

### 3.1 Key Findings Regarding Comparison Between SOUND Trial and Real‐World Data

Comparison between our real‐world patient cohort fulfilling the SOUND eligibility criteria and the SLNB arm of the SOUND trial demonstrates substantial similarity in key clinicopathological variables, supporting eligibility criteria and applicability of its findings to routine clinical populations. The age distribution was comparable (median 63 vs. 60 years), although the number of patients under the age of 50 years in SOUND trial was higher than in real‐world data (8.9% vs. 17.5%). In Finland, breast cancer screening is initiated at the age of 50, and the finding may reflect screening‐related differences. In pathological analysis, the tumor size was nearly identical in both cohorts (median 1.2 vs. 1.1 cm), but small tumors (T1a) were overrepresented in the SOUND trial compared to our real‐world data (10.0% vs. 4.1%). However, there were no differences in pathological axillary status, as 94.1% of patients in the real‐world cohort and 97.7% in the SOUND trial had 0–1 positive SLNs. The characteristics of breast cancer tumors, such as tumor grade and HR and HER2 status, were largely comparable to the real‐world data, indicating comparable oncological risk profiles. Differences in the proportions of PgR‐negative tumors (8.1% vs. 15.3%) and those with high proliferation (Ki‐67 ≥ 20%) may suggest that trial participants were more selectively enriched for biologically favorable disease, although these differences are likely of limited clinical relevance.

### 3.2 Key Findings Regarding Comparison Between INSEMA Trial and Real‐World Data

In contrast, patients eligible for the INSEMA trial in the real‐world cohort differed substantially from those enrolled in the trial. The age distribution was broadly comparable in both groups, with most patients between 50 and 70 years of age, reflecting the typical age range of screen‐detected breast cancer. However, tumor size differed substantially: the proportion of pT2 tumors was higher in the real‐world cohort (26.3% vs. 19.4%). This probably contributes to the greater proportion of pN1 cases (18.6% vs. 11.4%) as well, since larger tumors are known to have a higher propensity to generate metastasis in axilla. Additionally, the extent of nodal involvement varied: in our cohort, 3.4% of the patients had ≥ pN2 disease, compared to only 0.8% in the INSEMA SLNB arm, suggesting that patient selection in clinical practice may be less restrictive than in the controlled trial setting. Biological differences were also pronounced: although most tumors in both cohorts were hormone receptor–positive, the prevalence of HR+/HER2− tumors was much lower in the real‐world cohort (86.5% vs. 95.4%) and HER2‐positive and triple‐negative breast cancers were more common (HER2+: 8.1% vs. 3.3%; TNBC: 4.7% vs. 1.3%). Furthermore, in real‐world data, high‐proliferation tumors (Ki‐67 > 20%) were nearly three times more frequent (33.5% vs. 13.1%) than in the trial and the proportion of grade III tumors was also substantially higher (23.4% vs. 3.6%), indicating a more biologically aggressive disease spectrum.

### 3.3 Risk of pN2+ Disease

In the SOUND trial, only 4 patients (0.6%) out of a total of 708 patients had pN2+ disease. In the corresponding real‐world data, the risk of pN2+ disease was 1.5%. In the INSEMA trial, patients with positive axillary lymph nodes underwent second randomization to ALND or surveillance, and the pN2+ data are available only for the proportion of pN+ patients. Overall, 6.6% (253/3854) of all patients underwent ALND, and in these patients, 33 (13.0%) presented with pN2+ disease. In the overall real‐world cN0 patient population, the risk for pN2+ disease was 4.6%.

## 4 Discussion

The purpose of this study was to evaluate the external validity and real‐world applicability of the eligibility criteria of the SOUND and INSEMA trials. This issue is critical for the safe implementation of de‐escalation strategies, as differences between trial populations and real‐world patients may influence the generalizability of trial findings.

The present study demonstrates that the characteristics in the SLNB arm of the SOUND trial generally represent real‐world data well. However, it must be emphasized that the absolute number of patients in certain subgroups is rather low, as only 43 patients with triple‐negative breast cancer and 48 patients with HER2‐overexpressing tumors were included. Additionally, the exclusion criteria of the SOUND trial included the presence of multiple doubtful or suspicious lymph nodes and extensive multifocality or multicentricity. These exclusion criteria appear somewhat arbitrary, and the effect of these criteria is difficult to estimate, but it appears that this has not led to selection bias in patient enrollment. Despite these shortcomings, the findings of the present study suggest that the SOUND trial population closely reflects real‐world patients meeting its eligibility criteria and support the applicability of the SOUND trial's conclusions regarding the omission of SLN at least in Luminal subtype breast cancer.

In contrast, the study population in the INSEMA trial seems to represent biologically less‐aggressive breast cancer tumors compared to real‐world data. The real‐world INSEMA‐eligible population had a higher proportion of higher‐risk features, including larger tumors, higher tumor grade, higher Ki‐67 proliferation index, and a greater proportion of node‐positive disease, while the INSEMA trial population was more strongly enriched for low‐risk tumors, including a higher proportion of Grade I tumors and hormone receptor–positive, HER2‐negative disease.

Collectively, these differences suggest that real‐world patients represent a more anatomically and biologically heterogeneous population compared to the INSEMA trial's SLNB cohort. This highlights the need for caution when considering broader omission of SLNB in other than luminal subtypes of breast cancer.

### 4.1 Implications on Adjuvant Therapy

One of the main concerns regarding the omission of axillary surgery is that the detection of axillary metastases may guide decisions about adjuvant therapy. If the SLN is not examined, the treatment team must rely on other risk factors—such as tumor size, grade, biological markers like proliferation index, and multigene assays—to determine the need for adjuvant therapy. This may lead to either under‐ or overtreatment: some patients may be undertreated despite harboring occult nodal metastases, while others may receive chemotherapy unnecessarily due to an overestimation of risk.

Lymph node metastases remain one of the strongest prognostic factors in breast cancer and a key determinant in chemotherapy decision‐making. However, a recent study has shown that in postmenopausal women with hormone receptor–positive breast cancer who will be initiated hormonal treatment, chemotherapy may not be necessary for cN+ patients when treatment is guided by a recurrence score derived from the 21‐gene breast cancer assay. Thus, at least in this patient subgroup, axillary surgery is not essential for determining the need for adjuvant chemotherapy [16]. NCCN and ASCO guidelines emphasize that SLN biopsy should not be omitted if the information it provides could influence the use of adjuvant chemotherapy. Thus, omission of SLNB is feasible only when it is already clear in advance that the patient will either receive chemotherapy regardless of nodal status—or conversely, will not receive it under any circumstances [11, 17].

In HER2‐positive breast cancer, trastuzumab, and when indicated, pertuzumab and other antibody‐based therapies, have become cornerstones of adjuvant treatment. For small (< 1 cm) HER2‐positive tumors with negative lymph nodes, a de‐escalated regimen consisting of single‐agent chemotherapy (paclitaxel) combined with trastuzumab has been shown to be both effective and less toxic. However, in cases of more extensive disease or higher risk based on nodal involvement, a more intensive dual HER2 blockade (trastuzumab + pertuzumab) over a 12‐month period is recommended. Without SLNB, a critical component of risk stratification in HER2‐positive disease is lost: a patient who harbors occult lymph node metastases cannot be distinguished from the ones with truly node‐negative disease [18].

In hormone receptor–positive breast cancer, endocrine therapy (e.g., tamoxifen and/or an aromatase inhibitor ± ovarian function suppression) is currently recommended for all patients who are likely to benefit from the treatment regardless of nodal status. Therefore, omission of SLN biopsy typically does not affect whether a patient receives endocrine therapy, as this decision is based on ER/PgR status and overall clinical risk. The standard treatment duration for endocrine therapy is 5 years but extending the duration to 7–10 years further reduces the risk of disease recurrence and improves survival. This is particularly evident in patients with higher‐stage cancers, for example, N+, especially postmenopausal women. This suggests that nodal status may also influence the recommended optimal treatment duration [19, 20]. In addition, recent studies have shown that adjuvant treatment with ribociclib is indicated for all node‐positive luminal breast cancers, and abemaciclib is recommended when the patient has four or more (pN2+) metastatic lymph nodes [21, 22].

According to our findings, the risk of pN2+ disease in clinically node‐negative (cN0) patients is approximately 4.6% when no trial inclusion criteria are applied. However, the inclusion criteria of the SOUND and INSEMA trials efficiently exclude patients at higher risk. Further studies are warranted to better identify patients who may carry a higher‐than‐average risk of pN2+ disease and to determine the significance of omitting axillary surgery in these patients.

### 4.2 Implications for Clinical Practice

Since 2016, the Choosing Wisely campaign has recommended to avoid SLNB in women over 70 years of age with hormone receptor–positive pT1 tumors, based on earlier studies demonstrating minimal clinical benefit in this subgroup [23]. Nevertheless, registry data have shown that SLNB is still performed in over 80% of patients in this group in the United States [24]. One of the most significant barriers to implementing the omission of SLNB is concern about the generalizability of the results obtained from trials. To add confidence, real‐world data are essential in convincing the physicians in adopting the new treatment regimens to clinical practice. This study demonstrates that although the patient populations in the SOUND and INSEMA trials may not fully represent real‐life data, in small (< 2 cm) luminal breast cancers—which account for a large proportion of all breast cancers—the omission of axillary surgery appears to be applicable primarily to patients with small luminal breast cancers, as supported by previous randomized trials and by the similarity between trial populations and real‐world patients in this subgroup.

Axillary staging with imaging tools in lobular breast cancer is known to be less accurate than in ductal breast cancer due to the diffuse, single‐cell‐like growth pattern of lobular breast cancer. This may raise concerns about the safety of omitting SLNB in patients with lobular breast cancer in the absence of reliable imaging‐based assessment of axillary nodal status. Especially for the detection of pN2+ disease, MRI might be the most sensitive imaging tool but is not used for routine screening [25]. As only approximately one‐tenth of patients in the SOUND and INSEMA trials had lobular histology, the safety of SLNB omission in this subgroup remains uncertain. Further research on this subject is also warranted.

### 4.3 Other Studies

In a similar study to ours, the SOUND trial's SLNB arm was compared to breast cancer patients in Bahrain and there were found to be more premenopausal women and higher proportion of aggressive tumors in the study cohort in Bahrain than in the SOUND trial [26]. However, in Western populations, the populations and the tumor characteristics in the SOUND trial's SLNB arm seem to represent real‐world data well [27]. The results of other randomized trials regarding the safety of omitting SLNB in patients with cN0, T1‐2 invasive breast cancer undergoing breast conserving surgery, are to be expected in the upcoming years, including trials like the BOOG 2013‐08 and NAUTILUS [28, 29].

### 4.4 Limitations

As with any retrospective study, a limitation of this study is selection bias. The data used in this study were collected from one university hospital in one country with certain treatment guidelines and practices that might differ from those of other hospitals and countries. Multiple different clinicopathological characteristics of the patients were collected. However, one of the limitations of this study is the absence of certain variables, such as ethnicity. As the patient data consists of patients from only one university hospital district, it only represents the population of that area. The study could be expanded by studying data from several districts in different countries which could then generate more information that could be applied to more clinics with different kinds of populations.

The data in this study were collected retrospectively, and as a result, information on menopausal status and, in a significant proportion of cases, BMI is missing. However, menopausal status strongly correlates with patient age, and BMI data were available for a substantial portion of the cohort, making it unlikely that these limitations significantly affect the generalizability of the study findings. While patient populations may vary between clinical settings, an essential aim of this study is precisely to assess the extent to which the results of previous RCTs can be generalized to real‐world data.

Furthermore, as this study was designed to assess external validity rather than oncologic outcomes, no recurrence or survival analyses were performed. However, evaluating eligibility criteria and clinicopathologic characteristics represents a critical step in determining the generalizability and appropriate clinical implementation of trial findings.

## 5 Conclusion

The population included in the SOUND trial is generally representative of real‐world patients, although certain subgroups, such as those with triple‐negative or HER2‐positive breast cancer, were underrepresented. In contrast, the INSEMA trial population appears to represent a more selected subgroup with biologically less aggressive disease, while real‐world patients demonstrate greater anatomical and biological heterogeneity.

The findings of the present study support the real‐world applicability and generalizability of the SOUND and INSEMA eligibility criteria primarily in small (< 2 cm) luminal breast cancers. Further research needed to evaluate the applicability of SLNB omission in other biological subtypes and higher‐risk patient groups.

## Funding

The authors have nothing to report.

## Ethics Statement

This research study was conducted retrospectively from data obtained for clinical purposes. The research protocol of the study was approved by the Southwest Finland Wellbeing Services County (2024‐0978‐OP). No ethical approval was required for this retrospective study.

## Conflicts of Interest

The authors declare no conflicts of interest.

## Data Availability

The data that support the findings of this study are available on request from the corresponding author. The data are not publicly available due to privacy or ethical restrictions.
